# Supplementary material for: Trypanosoma cruzi Presenilin-Like Transmembrane Aspartyl Protease: Characterization and Cellular Localization
Source: Biomolecules. 2020 Nov 17;10(11):1564. doi: 10.3390/biom10111564 (PMC7698364; doi:10.3390/biom10111564)
Supplement: Supplementary file 1 [file biomolecules-10-01564-s001.pdf]

Supplementary Material

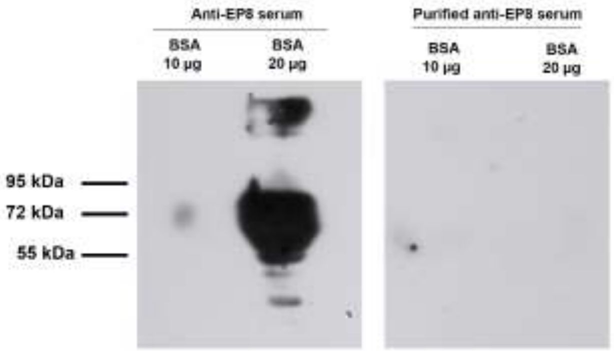

Figure S1: Western blot analysis of rabbit anti-EP8 depleted of anti-BSA antibodies by a Sepharose-BSA column. SDS-PAGE (10%) of BSA (10 and 20 µg) under reducing conditions, revelated by chemiluminescence using anti-EP8 peptide polyclonal rabbit sera before and after purification in Sepharose-BSA column.

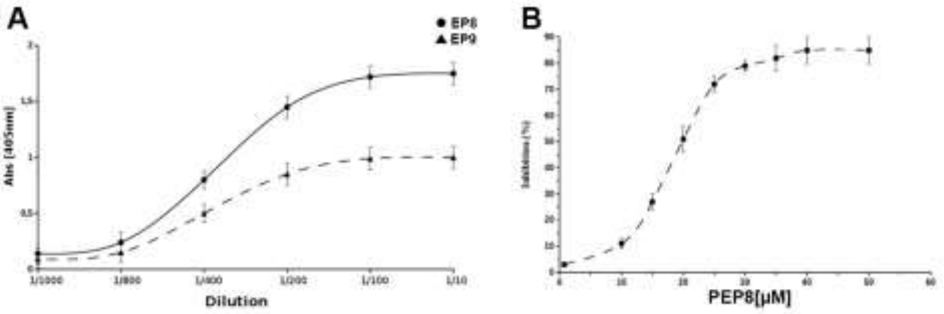

Figure S2: Performance and specificity of the rabbit sera anti-synthetic peptide EP8 and EP9. Two individual rabbits were immunized with either synthetic peptide EP8 or EP9 and serum collected on day 42. Sera sensitivity was evaluated by ELISA using a dilution series (Panel A; ● Rabbit #1, EP8 and ▲ Rabbit #2, EP9). Due to the higher sensitivity of the anti-EP8 serum, a competition assay was performed with increasing concentrations of purified peptide (Panel B; 0.75 µM

to 50 μM). The percent inhibition of binding was calculated with 100% as the value in the absence of competing peptide.

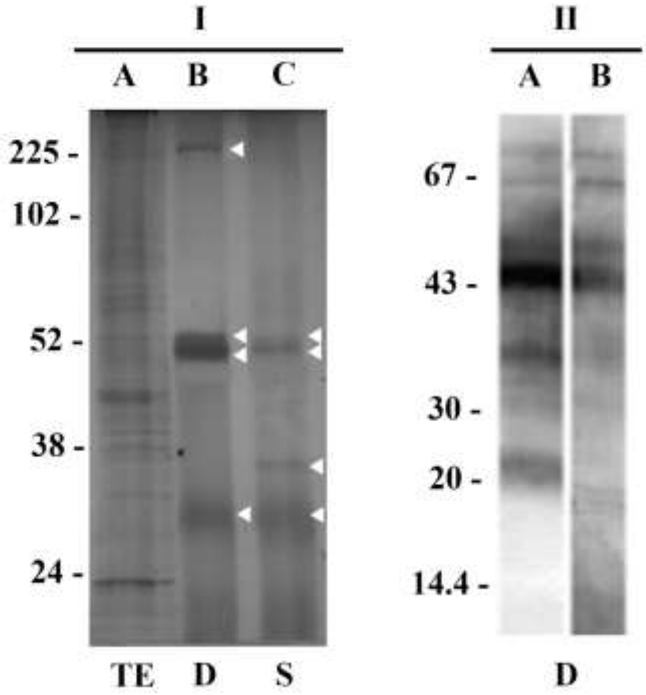

Figure S3: SDS-PAGE (I) and western blot (II) of T. cruzi epimastigote detergent (S) and soluble (S) fractions. Analysis by SDS-PAGE and silver staining of the proteins of the total extract (TE), detergent (D) and soluble (S) fraction of T. cruzi epimastigotes obtained using the methodology described previously [22] and purified by pepstatin-Agarose column (Panel I). Panel II show western blotting analyzes of the affinity column purified fractions reacting with anti-detergent fraction immunized rabbit (A) and pool of sera from chagasic patients (n = 5) (B). Panel I analyzed about 25 μg /slot and panels II (about 70 μg/slot). The antibody reactivities were revealed with goat anti-human and anti-rabbit IgG peroxidase-conjugated, followed by incubation with H_2O_2 and 3,3′-diaminobenzidine. The white arrows indicate the position of main bands in the detergent and soluble fractions. Molecular mass values of standard proteins (Mr x 10^3) are indicated in the left.

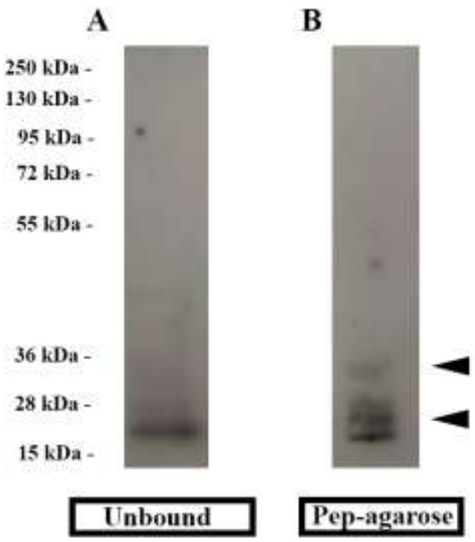

Figure S4: *T. cruzi* PS-like binds to pepstatin-agarose. Parasite lysates (40 μg) were adsorbed onto pepstatin-agarose, and the proteins that remained bound were analyzed by Western blotting with anti-EP8 sera. Panel A: Correspond to the total cell lysate unbound to pepstatin-agarose after incubation for 24 hours at 4ºC in STE buffer. Panel B: Proteins bound to pepstatin-agarose revealed by anti-EP8 sera. Note that PS-like bands corresponding to ~24 kDa and ~32 kDa (arrowhead), also identified in total extract, were absorbed to pepstatin agarose.

Formatado: Fonte: Não Negrito

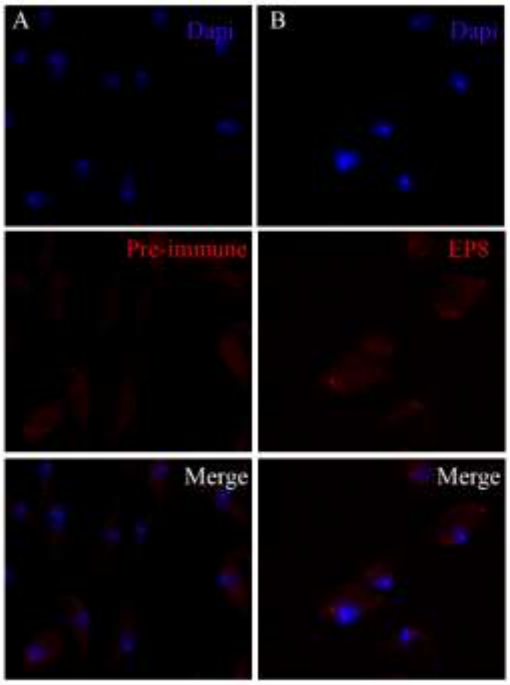

Figure S5: Immunofluorescent localization of *T. cruzi* PS-like protein in *T. cruzi* epimastigotes. Parasites were cultivated in BHI medium supplemented with 10% FBS, using rabbit anti-EP8 sera and pre-immune sera (A) Parasites were incubated overnight with pre-immune sera (red, Panel A), anti-EP8 (red, Panel B).

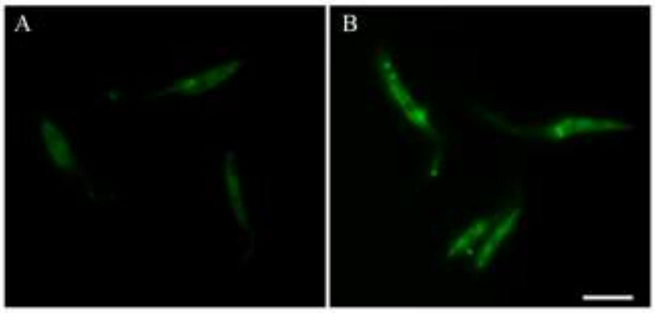

Figure S6: Autophagic vacuoles labeled with monodansylcadaverine (MDC) increase during epimastigotes serum deprivation. (A) Labeling of autophagic vacuoles in epimastigotes cultivated in BHI medium supplemented with 10% FBS under standard conditions. (B) Epimastigotes incubated in BHI medium without FBS for 24 hours, showing numerous of MDC-labeled vesicles. Scale bar = 10 μm.
